# tDCS-induced enhancement of cognitive flexibility in autism: role of frontal lobe and associated neural circuits

**DOI:** 10.3389/fnbeh.2025.1631236

**Published:** 2025-08-12

**Authors:** Yanan Han, Anqin Dong, Chenyi Xia, Zhe Zhang, Wenjing Hu, Tingli He, Xinxin Cui, Chengming Xu, Hongyan Xu, Zhangying Zhou, Danmeng Cheng, Shuo Zhang, Liguo Li, Youcai Tang, Pengyuan Zheng, Xianwen Dong

**Affiliations:** ^1^Henan Provincial Key Laboratory of Rehabilitation Medicine, The Fifth Affiliated Hospital of Zhengzhou University, Zhengzhou, Henan, China; ^2^Institute of Rehabilitation Medicine, Henan Academy of Medical Sciences, Zhengzhou, Henan, China; ^3^School of Basic Medical Sciences, Shanghai University of Traditional Chinese Medicine, Shanghai, China

**Keywords:** autism spectrum disorder, cognitive flexibility, tDCS, dorsolateral prefrontal, hippocampal, striatum

## Abstract

**Background:**

Autism spectrum disorder (ASD) is a neurodevelopmental disorder characterized by impaired social interaction and restricted, repetitive behaviors (RRBs). These symptoms may stem from cognitive flexibility deficits, with dysfunction in the prefrontal cortex (PFC) and related neural circuits proposed as underlying mechanisms.

**Objectives:**

This study examined whether transcranial direct current stimulation (tDCS) could enhance PFC activity and functional connectivity, thereby improving cognitive flexibility in a valproic acid (VPA)-induced ASD rat model.

**Methods:**

Pregnant Sprague-Dawley rats were administered VPA (600 mg/kg, E12.5) or saline. VPA-exposed offspring exhibiting curved tails received tDCS and underwent behavioral tests, including the three-chamber social interaction test and cross-maze rule-shifting task, while local field potentials (LFPs) were recorded. Immunohistochemistry was performed to evaluate microglial activation (Iba1 +) and synaptic density (PSD95).

**Results:**

Valproic acid -exposed offspring displayed significant social interaction deficits and impaired cognitive flexibility, alongside disrupted functional connectivity in frontal-striato-hippocampal circuits. Neuroinflammatory analysis revealed elevated Iba1+ microglial density (*p* < 0.05) and increased PSD95 expression (*p* < 0.05). After tDCS intervention, VPA rats exhibited restored sociability and cognitive performance, normalized functional connectivity, and significantly reduced microglial activation (*p* < 0.05), though PSD95 levels were unaffected.

**Conclusion:**

Our results indicate that tDCS ameliorates ASD-like phenotypes in VPA rats, potentially through microglial suppression and PFC network synchronization. These findings support neuromodulation as a promising therapeutic approach for ASD-related cognitive dysfunction.

## 1 Introduction

Autism spectrum disorder (ASD) is a heterogeneous group of genetic neurobehavioral disorders characterized by impaired social interaction and communication, repetitive and restricted behaviors (RRBs) and limited interests (Genovese and Butler, 2023; Won et al., 2012). The global prevalence of ASD has risen significantly in recent decades, currently affecting approximately 1% of the population (de Araujo, 2022). Although the exact etiology remains unclear, ASD likely arises from a complex interplay of genetic, epigenetic, and environmental factors (Bhandari et al., 2020). Notably, prenatal valproic acid (VPA) exposure has been identified as a significant environmental risk factor for ASD (Zarate-Lopez et al., 2024). Clinical studies demonstrate that first-trimester VPA exposure substantially increases ASD risk in humans (Taleb et al., 2021), while animal studies show that VPA-exposed offspring develop structural, behavioral, and molecular abnormalities remarkably similar to those observed in ASD patients (Luo et al., 2023; Triyasakorn et al., 2022). Currently, ASD animal models mainly consist of two types: environmentally induced (via chemical exposure or immune activation) and genetically engineered models (Ergaz et al., 2016). Among these, the VPA-induced model has emerged as one of the most widely used and validated paradigms, exhibiting robust face, construct, and predictive validity (Nicolini and Fahnestock, 2018). Importantly, this model may better recapitulate idiopathic ASD cases of suspected environmental or epigenetic origin compared to transgenic models targeting single ASD-associated genes (Nicolini and Fahnestock, 2018), making it particularly valuable for investigating the neurobiological mechanisms underlying ASD.

A hallmark feature of ASD is impaired cognitive flexibility—the ability to adapt behavior in response to changing environmental demands (Seifried et al., 2023). This deficit is considered a key contributor to the RRBs and limited interests characteristic of ASD (Dajani and Uddin, 2015; Geurts et al., 2009), as individuals struggle to shift attention or adjust strategies when confronted with novel situations (Dajani and Uddin, 2015; Lage et al., 2024). As a core executive function mediated by prefrontal cortex (PFC) circuits involving the striatum and hippocampus (Logue and Gould, 2014), cognitive flexibility is particularly compromised in ASD. Electrophysiological evidence indicates that increased theta power during cognitive tasks correlates with greater cognitive flexibility (Tan et al., 2024). However, children with ASD show attenuated frontal theta power increases during cognitive flexibility tasks (e.g., the Wisconsin Card Sorting Test) (Yeung et al., 2016), reflecting underlying neural dysfunction. Importantly, cognitive inflexibility and social impairments may interact bidirectionally, highlighting this domain as a crucial therapeutic target.

The cognitive flexibility impairments observed in ASD appear to stem from structural and functional abnormalities in the PFC and its associated networks. As the neural substrate for higher-order cognition (Xu et al., 2019), the PFC demonstrates widespread alterations in ASD, encompassing structural, physiological, and functional domains (Just et al., 2004; Mundy, 2003; Teffer and Semendeferi, 2012). The frontostriatal circuit, supporting adaptive behavior (Fetcho et al., 2023), is particularly affected, with downstream targets (the dorsal striatum and nucleus accumbens) playing distinct roles in behavioral flexibility (Welch et al., 2007). The frontostriatal synaptic plasticity is modulated by dopaminergic projections from the ventral tegmental area (Yawata et al., 2012), and disruptions in this circuit are associated with cognitive rigidity (Lin et al., 2024) and stereotyped behaviors (Welch et al., 2007). Furthermore, novelty-induced resetting of hippocampal-frontal circuits facilitates learning-related plasticity (Park et al., 2021), a process that may be disrupted in ASD.

Emerging evidence indicates that ASD is characterized by insufficient asymmetric long-range connectivity, particularly between the frontal lobes and other regions. Individuals with ASD exhibit reduced asymmetric connectivity between the medial PFC, posterior cingulate cortex, and other regions (Long et al., 2016), as well as weaker frontal-occipital connectivity that correlates with symptom severity (Barttfeld et al., 2011). Notably, individuals with ASD demonstrate compromised top-down regulatory control from frontal cortical regions over subcortical systems (Horwitz et al., 1988). Genetic evidence from Shank3B−/− mouse models further demonstrates that synaptic dysfunction directly impairs prefrontal and frontostriatal circuitry, with these connectivity deficits directly correlating with socio-communicative impairments (Pagani et al., 2019). Collectively, these findings strongly implicate disrupted PFC-mediated long-range connectivity as a neural substrate for cognitive inflexibility in ASD, highlighting promising targets for neuromodulation-based therapies.

At the cellular level, ASD pathophysiology involves prominent neuroinflammatory mechanisms characterized by microglial hyperactivation and synaptic impairment (Vargas et al., 2005). Overactivated microglia secrete pro-inflammatory cytokines and reactive oxygen species, potentially compromising synaptic plasticity and neuronal function (Leng and Edison, 2021). These pathological alterations are especially evident in the PFC, where dysregulated expression of synaptic proteins, particularly elevated levels of postsynaptic density protein 95 (PSD95), may contribute to broader network dysfunction (Luo et al., 2023).

Transcranial direct current stimulation (tDCS) is a non-invasive neuromodulation technique that alters cortical excitability by applying low-intensity direct current (usually no more than 2 mA) to targeted brain regions (Qiu et al., 2021). The primary mechanism involves polarity-dependent modulation of resting membrane potentials, with cathodal stimulation inducing hyperpolarization and anodal stimulation causing depolarization. These aftereffects resemble synaptic long-term facilitation (LTF) mechanisms. Specifically, anodal tDCS modulates transmembrane potentials to influence calcium and sodium ion concentrations, thereby activating NMDA receptors and enhancing long-term potentiation (LTP) (Roche et al., 2015). The effects of tDCS on resting-state network activity have been investigated. For instance, anodic stimulation of the left motor cortex enhances its functional connectivity with the ipsilateral thalamus, caudate nucleus and parietal association cortex, while cathodal stimulation reduces connectivity with the contralateral thalamus. Bilateral stimulation of the motor cortex induces widespread connectivity changes, particularly affecting PFC and primary or secondary motor cortical networks (Ethridge et al., 2022; Grami et al., 2021; Liu et al., 2021; Tang et al., 2023). Based on these findings, we examined frontal-hippocampal-striatal functional connectivity following anodal stimulation in a murine model.

The dorsolateral prefrontal cortex (DLPFC), a key region for executive functions and cognitive control (Kong et al., 2019; Tao et al., 2017; Xia et al., 2021), has gained recognition as a promising neuromodulation target for addressing cognitive and social impairments in ASD (Chen et al., 2024; Gandhi and Lee, 2020; Li et al., 2022; Qiu et al., 2021). Anodal tDCS targeting DLPFC demonstrates significant efficacy in enhancing various cognitive domains including memory (Ferrucci and Priori, 2014), attention (Nitsche et al., 2008), learning (Coffman et al., 2014) and connectivity or plasticity (Tu et al., 2021). This therapeutic potential is further supported by well-characterized DLPFC abnormalities in ASD populations, which encompass both structural alterations [increased neuronal density (Courchesne et al., 2011)] and functional deficits [regional hypoactivity (Carlisi et al., 2017)]. These pathological alterations collectively contribute to core ASD symptoms such as emotional processing deficits (Nejati et al., 2021), executive dysfunction (Friedman and Robbins, 2022) and language impairments (Yeh et al., 2024). The therapeutic mechanisms of DLPFC stimulation may involve modulation of γ-aminobutyric acid (GABA) levels (Bunai et al., 2021) and normalization of cortico-striatal circuits implicated in ASD pathogenesis (Soghomonian, 2023). Supporting this therapeutic strategy, tDCS has demonstrated cognitive flexibility improvements in other clinical populations through excitability and enhancement of functional connectivity (multiple mechanisms, including attenuation of frontal glial-mediated inflammation, regulation of regional Bunai et al., 2021; Liu et al., 2021; Pikhovych et al., 2016), all of which may similarly ameliorate core cognitive deficits in ASD.

Based on these findings, we investigated whether tDCS could improve cognitive flexibility in a VPA-induced rat model of ASD. We hypothesized that tDCS would attenuate cognitive inflexibility by reducing neuroinflammation responses, enhancing synaptic function and restoring functional connectivity within frontal-striato-hippocampal circuits. Through a multimodal approach combining behavioral assessments, electrophysiological recordings, and immunohistochemical analyses, this study provides new evidence supporting tDCS as a potential treatment for ASD-related cognitive impairments while elucidating the underlying neural mechanisms.

## 2 Materials and methods

### 2.1 Animals

Sprague-Dawley (SD) rats were provided by Zhengzhou Huaxing Experimental Animal Breeding Farm and housed under standard laboratory conditions (24 ± 0.5°C, 50 ± 5% humidity) with a 12 h light/dark cycle (light on 7 a.m.–7 p.m.). Food and water were provided *ad libitum*. Two female rats were housed with one male per cage. Female rats were examined for the presence of a vaginal plug, which was used to define embryonic day 0.5 (E0.5). On embryonic day 12.5 (E12.5), pregnant dams were randomly assigned to either the VPA or control group. The VPA group received a single intraperitoneal injection of 600 mg/kg sodium valproate, while control animals received an equivalent volume of saline. Based on well-documented sex-specific effects of prenatal VPA exposure in inducing ASD-like phenotypes (Nicolini and Fahnestock, 2018), only male offspring were included in subsequent experiments. Offspring from saline-injected dams served as the normal control group (CON, *n* = 10). VPA-exposed offspring were screened for physical abnormalities (e.g., tail curvature) and randomly allocated into two groups: the untreated VPA model group (VPA, *n* = 10) and the tDCS intervention group (tDCS, *n* = 10). The experiment was approved by the Medical Ethics Committee of the Fifth Affiliated Hospital of Zhengzhou University.

### 2.2 Electrode implantation and tDCS intervention

Electrodes were surgically implanted in all three experimental groups when the rats reached 6 weeks of age (Ramanathan et al., 2018). The electrodes were fixed on the cranial surface using denture base tray resin. After recovery from electrode implantation surgery to week 8, rats in the tDCS group received tDCS in a ramp on/off period (a gradually increasing current, rather than an immediate adjustment to the target value). This stimulation was provided using a direct current power supply (MAISHENG, MS-152D, Dongguan, China) with a sampling rate of 10 kHz and a filter setting of 100 Hz. And 100 μA direct current stimulation was administrated at the left DLPFC of the rats for 20 min/d for 4 consecutive days, with an interval of 2 days, and then stimulation for 4 days. The remaining groups received sham stimulation (connected to a direct current power but not energized) for the same duration of the session. In summary, electrodes were implanted in littermates at week 6, tDCS was administrated to littermates in the tDCS group at week 8, and behavioral tests were conducted at week 10.

### 2.3 Three-chamber social interaction test

Three-chamber social interaction test (Arakawa, 2023; Park et al., 2023; Zhou and Jia, 2021) (TCT) was used to assess the social interest and social cognition of rats. The three-chamber social behavior box (120 cm × 80 cm × 80 cm) is a box made of PVC material divided into three compartments with two cylindrical metal cages. The tested rats were placed in the central compartment and allowed to freely explore the three compartments for 10 min (stage 1). Then an empty metal cage (E) and an age-matched strange male rat (S1) were placed symmetrically into the two side compartments, respectively, and the tested rats were placed into the central compartment to explore freely for 10 min (stage 2). The time that the tested rats sniffed S1 and E (sniffing was defined as the head of the tested rat being within 2 cm of the wire cage) was measured. Another age-matched, unfamiliar rat (S2) was placed in the previously empty metal cage, and the tested rat was again placed in the middle compartment to explore freely for 10 min (stage 3). The time the tested rat spent sniffing S1 and S2 was measured. We calculated the social interaction index at stage2 and social novelty preference index at stage3. Stage2: social interaction index = (S1−E)/(S1 + E) × 100%, Stage3: social novelty preference index = (S2−S1)/(S2 + S1) × 100%.

### 2.4 Repetitive and restricted behavior tests

We conducted Marble burying test and Wood chew test as previously reported (Kim et al., 2014; Sacai et al., 2020; Wong et al., 2020). Please see the Supplementary Methods for details regarding tests.

### 2.5 Anxiety behavior test

Open field test (Sturman et al., 2018) was used to assess the level of anxiety in rats (Zhang et al., 2022). Please see the Supplementary Methods for details regarding test.

### 2.6 Novel object recognition

Novel object recognition (NOR) (Hernandez-Rapp et al., 2015; Wooden et al., 2021) test was performed in order to evaluate novelty preferences and object exploration behavior in rodents. Rats were placed in the center of the device (80 cm × 80 cm × 50 cm) and adapted freely for 5 min (stage 1). Identical objects (A and B) were placed in the left and right positions of one side of the arena at equal distances from the side walls. The rats were placed in the center of the device and allowed to explore freely for 5 min, and then the rats were taken out for 1 h (stage2). An original object (B) was replaced with a new object (C) of different shape and color, and the rats were again placed in the arena and allowed to explore freely for 5 min (stage3). The time spent exploring objects (B and C) was counted (object exploration was defined as the rat’s nose being no more than 2 cm away from the object or touching the object with its nose). The novel object recognition index was calculated. Novel object recognition index or discrimination index = explore New Object C time/(Explore Old Object B time + Explore New Object C time).

### 2.7 Cross-maze rule-shifting task

**FIGURE 1:**
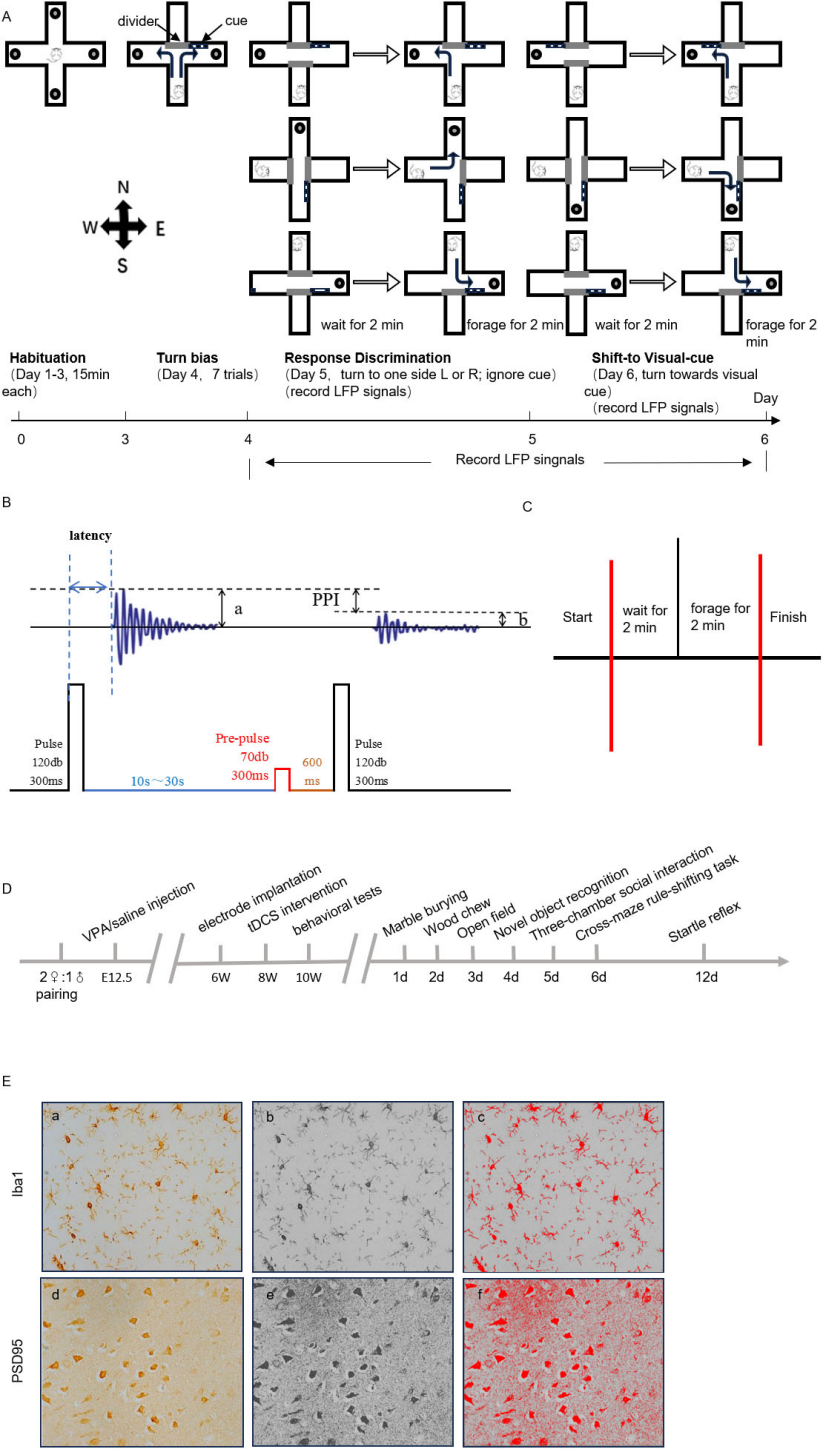
**(A)** Schematic diagram of the experimental flow of the Cross-maze rule-shifting task (CMRST). **(B)** Schematic diagram of startle reflex waveform. **(C)** Local field potential (LFP) recordings of CMRST response discrimination stage and shift-to visual-cue stage. **(D)** Experiment diagram. **(E)** Representative immunohistochemical (IHC) staining images. (a) Immunohistochemical staining of Iba1-positive cells. (b) The 8-bit grayscale image. (c) Image after automatic threshold adjustment (400 × ). (d) PSD95 immunohistochemical staining image. (e) 8-bit grayscale image. (f) Image after automatic threshold adjustment (400 × ).

The Cross-maze rule-shifting task (CMRST) (Driskill et al., 2022) is used to test decision-making capabilities and the ability to inhibit a prepotent but inappropriate response in training for a rule switching task, thereby assessing cognitive flexibility. The maze (42.5 cm × 15 cm × 35 cm) was set up in a room with no visual cues and tested at the same time every day. The experiment was divided into four stages. First, rats were habituated to the maze for 3 days, during which all arms were baited with milk tablet and animals were allowed to freely explore the maze for 15 min (stage 1). Then, a divider was used to seal one of the arms and milk tablets was placed into each of the two arms. A black and white striped visual cue was randomly placed near the entrance to one of the entry arms and rats were placed in the stem arm and allowed to turn left or right to obtain the milk tablet. After the mouse found and ate the food rewards, it was placed back into the stem arm until it turned to the other arm and found the food rewards and a total of seven trails were run (stage 2). A divider was used to seal one of the arms and always place a milk tablet on the left arm. The black and white striped visual cue was randomly placed near the entrance to one of the arms. Each rat was put into the stem arm separated by divider in the cross maze, and the divider was removed after 2 min. It was correct for the rat to choose to enter the left arm within 2 min for the first time and find the food rewards. The location of the stem arm was rotated among three arms. The rats will enter the next stage when they made nine correct choices in any block of 10 trials in Response Discrimination (stage 3). Finally, rats were tested on the ability to shift their strategy and now required to learn to “follow the visual cue” in order to obtain milk tablets. The location of the visual cue and the position of the start arm were again varied. The training and response criteria for the Shift-to Visual-Cue Discrimination were identical to those during Response Discrimination except that the cue and milk tablet are on the same arm. Errors were scored when a rat entered the incorrect opposite arm on eight trials performed (stage 4) (Figure 1A).

### 2.8 Startle reflex

The startle reflex (Maamrah et al., 2023) is an autonomic nervous system response triggered by sudden and intense stimuli, characterized by rapid muscle contractions and behavioral responses. Pre-pulse inhibition (PPI) refers to the inhibition of startle reflex amplitude by weak stimulus given before startle reflex stimulation, which is a sensorimotor gated process of brain adaptation to complex environments. The startle reflex represents a well-established behavioral paradigm for evaluating stress responses, emotional states, and cognitive functions in rodents. In this standardized procedure, experimental rats were placed in the testing chamber and allowed sufficient acclimation time until baseline electrophysiological signals stabilized. The test protocol consisted of delivering an abrupt high-intensity acoustic stimulus (120 dB, 300 ms duration) to elicit the startle response, with behavioral reactions being recorded. For PPI assessment, a dual-pulse paradigm was employed following baseline stabilization, comprising a pre-pulse stimulus (70 dB, 300 ms) preceding the startle stimulus by 600 ms. All experimental procedures were conducted within a sound-attenuated chamber to ensure environmental control. To minimize habituation and fatigue effects, multiple trials were administered with adequate inter-trial intervals. Key parameters analyzed included PPI index, calculated as *(a–b)/a × 100%* and startle reflex latency (Figure 1B).

### 2.9 Electrophysiology recordings

In the Cross-maze rule-shifting task trial, local field potential (LFP) was captured during the response discrimination stage and shift-to visual-cue stage (Figure 1C). Functional connectivity refers to the coordinated synchronization of brain functions, which can be reflected by LFP Coherence (COH), and LFP data are mainly analyzed for power spectral density and coherence. The coherence metric [*COH_*xy*_(f)*] was calculated as the normalized cross-power spectral density *|S_*xy*_(f)|^2^/[S_*xx*_(f)⋅Syy(f)]*, where *S_*xy*_(f)* represents the cross-spectrum between signals x(t) and y(t), while *S_*xx*_(f)* and *S_*yy*_(f)* denote their respective auto-power spectra. The coherence value [COHxy(f)], ranges between 0 and 1. A value of 0 indicates no linear correlation between signals x(t) and y(t) at frequency f, whereas a value of 1 signifies a perfect linear relationship between the two signals at that frequency. *COH_*xy*_(f)* = *|K_*xy*_(f)|^2^* = *|S_*xy*_(f)|^2^* / [*S_*xx*_(f)S_*yy*_(f)*]. We provide the complete experimental flowchart (Figure 1D).

### 2.10 Immunohistochemistry

Brain tissues were processed after behavioral and electrophysiological tests in each group of animals, sectioned along the coronal plane of the brain at a thickness of 2–3 mm, and placed in disposable plastic dehydrated embedding boxes. The slides were dehydrated by ethanol and transparent by xylene at 25°C. Slides were dewaxed at 60°C for 1 h, rinsed with PBS buffer for 15 min and treated with 3% H_2_O_2_ solution for 10 min. Appropriate amount of primary antibody (Iba1 antibody: GTX100042, Anti-PSD95 Recombinant Rabbit Monoclonal Antibody: ET1602-20) was added to the slides. Then, sections were incubated with secondary antibody and labeling was visualized by DAB Substrate Chromogen solution. The degree of color development was observed by using a microscope.

The dorsolateral prefrontal region was identified. Iba1-positive cell expression (Figure 1E, a–c) and PSD95-positive expression (Figure 1E, e, f) was observed. The number of Iba1-positive cells and the Iba1-positive area in DLPFC were counted. The mean optical density, integrated optical density and positive area in DLPFC of PSD95 were calculated.

### 2.11 Statistical analysis

Data analysis was conducted using SPSS 23.0, NeuroExplorer5, PlexUtil, and Spike2, while GraphPad Prism 10.0 software was used to automatically generate graphs. Animal behavior videos were tracked and analyzed using Any-maze software. For continuous data that were normally distributed with uniform variance, the following statistical tests were applied: the *t*-test for two independent samples for comparisons between independent groups, the paired *t*-test for comparisons between paired groups, one-way analysis of variance (ANOVA) for comparisons among three groups, and Bonferroni test for *post hoc* comparisons between two groups. For continuous data that were non-normally distributed or exhibited variance, the Mann-Whitney U test was utilized for comparisons between two groups, while the Kruskal-Wallis H test was applied for comparisons among three groups. A *p*-value of < 0.05 was considered statistically significant.

## 3 Result

### 3.1 Social behavior test

**FIGURE 2:**
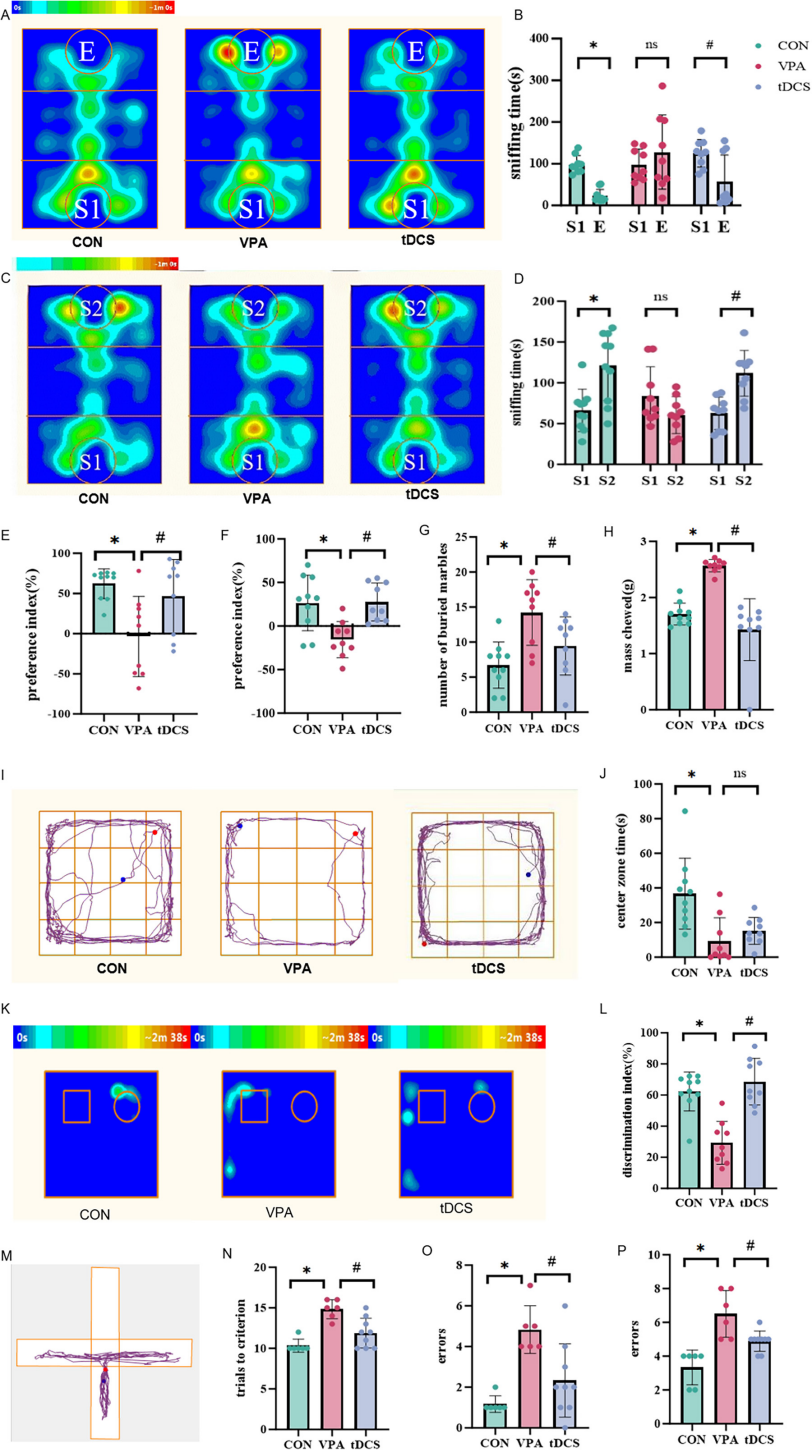
Results of behavioral tests. **(A–F)** Three-chamber social interaction test (TCT) results. **(A)** Heat map of tracking for each group of mice during stage 2. E: empty metal cage. S1: an age-matched strange male rat. **(B)** Comparison of sniffing time for rats toward S1 and E in each group during stage 2 [CON: df = 9, t = 9.201, *p* < 0.001; valproic acid (VPA): df = 8, t = –0.747, *p* = 0.477; transcranial direct current stim (tDCS): df = 8, t = 2.546, *p* = 0.034]. **(C)** Heat map of tracking for each group of mice during stage 3. S2: an unfamiliar male rat. **(D)** Comparison of sniffing time for rats toward S1 and S2 in each group during stage 3 (CON: df = 9, z = –2.293, *p* = 0.022; VPA: df = 8, t = 1.939, *p* = 0.088; tDCS: df = 8, t = –3.862, *p* = 0.005). **(E)** Comparison of the social preference index among the rat groups in stage 2 (df = 2, H = 7.449, *p* = 0.024). **(F)** Comparison of social novelty preference index among groups of rats in Stage 3 (df = 2, H = 11.366, *p* = 0.003). **(G)** A comparison was made of the number of marbles buried by each group of rats. VPA rats buried significantly more marbles (df _between_ _groups_ = 2, F = 8.259, *p* = 0.002). **(H)** A comparison was made of the mass of sticks chewed by each group of rats, revealing a significant increase in the VPA group (df = 2, H = 18.7, *p* < 0.001). **(I)** Tracking plots of open field trajectories for each group of rats, designating the four small cells in the center as the central area and the remainder as the peripheral area. **(J)** Comparison of the time taken by rats to enter the central region across groups. Rats in the VPA group spent less time exploring the central area, while those in the tDCS group showed a non-significant increase in central exploration time (df = 2, H = 12.265, *p* = 0.002). **(K)** A heat map depicting tracking of new and familiar objects for each group of rats during Stage 3. **(L)** Comparison of the novel object recognition index among the groups of mice (df = 2, H = 15.486, *p* < 0.001). **(M)** Trajectory plot of rats. **(N)** Number of trials required to reach the standard for each group of rats during Stage 3 (df = 2, H = 12.428, *p* = 0.002). **(O)** Number of errors made by rats in each group during Stage 3 (df = 2, H = 11.188, *p* = 0.004). **(P)** Number of errors in rats in each group during Stage 4 (df = 2, H = 14.230, *p* < 0.001). Data were analyzed by two-tailed paired *t*-tests **(B)**, Wilcoxon rank-sum test for the CON group and two-tailed paired *t*-tests for VPA and tDCS group **(D)**, one-way analysis of variance (ANOVA) **(G)** and Kruskal-Wallis H test **(E,F,H,J,L,N–P)**. *p < 0.05, #*p* < 0.05, ns, not significantly difference. *n* = 10 in the CON group, *n* = 9 in the VPA group and *n* = 9 in the tDCS group. But in the Cross-maze rule-shifting task (CMRST), the sample size of the CON group and VPA group reduced to *n* = 6 due to electrode detachment and electrode occlusion.

Following electrode implantation, one mortality occurred in each of the VPA and tDCS groups, resulting in final group sizes of 10 rats in the CON group, and nine rats each in the VPA and tDCS groups. The results from stage 2 of TCT indicate (Figures 2A, B) that CON rats significantly spent more time sniffing S1 compared to E (*p* < 0.05). There was no significant difference of sniffing time between S1 and E in VPA rats (*p* > 0.05). Following tDCS, rats spent more time sniffing S1 compared to sniffing E (*p* < 0.05). The social index results (Figure 2E) indicate that VPA rats had a lower social index compared to CON rats and spent less time sniffing S1 (*p* < 0.05). Rats in the tDCS group exhibited a higher social index compared to the VPA group and spent more time sniffing S1 (*p* < 0.05), suggesting that tDCS somewhat improved socialization deficits in offspring.

The results from stage 3 of TCT demonstrate that CON rats spent significantly more time sniffing S2 compared to S1 (*p* < 0.05), while VPA rats spent more time sniffing S1 than S2 (*p* > 0.05). After receiving tDCS, rats spent more time sniffing S2 than S1 (*p* < 0.05). The computed social novelty preference index results (Figures 2C, D, F) indicate that VPA rats exhibited a lower social novelty preference index compared to the CON group and spent less time sniffing S2 (*p* < 0.05). Rats in the tDCS group exhibited a higher social novelty preference index compared to VPA rats, spending more time sniffing S2 (*p* < 0.05). This suggests that tDCS somewhat improves social novelty exploration in rats exposed to VPA during pregnancy.

In summary, offspring in the VPA group exhibited deficits in social interaction, while the tDCS intervention showed potential to enhance social abilities and promote social novelty exploration to some extent.

### 3.2 Repetitive and restricted behavior test

The results of the marble burying test indicated that the number of marbles buried by VPA rats was significantly higher than CON rats (*p* < 0.05). The number of marbles buried by tDCS rats was reduced compared to the VPA group (*p* < 0.05). This suggests that tDCS has an ameliorative effect on the marble burying behavior of rats in the VPA group (Figure 2G).

The wood chew test results indicated that VPA rats chewed a greater mass of sticks than the CON group (*p* < 0.05). Furthermore, tDCS rats exhibited a significant reduction in the mass of sticks chewed compared to those in the VPA group (*p* < 0.05). These findings imply that tDCS may improve stick chewing behavior (Figure 2H).

### 3.3 Anxiety behavior test

The open field test results (Figures 2I, J) indicated that VPA rats spent less time in the central area than CON rats (*p* < 0.05). Furthermore, there was no significant increase in the time spent in the central area by tDCS rats compared to the VPA rats (*p* > 0.05). These results indicate significant anxiety in the VPA rats. Additionally, tDCS did not significantly alleviate anxiety in VPA rats.

### 3.4 Novel object recognition

The results indicated that the discrimination index decreased in VPA rats compared to the CON rats (*p* < 0.05). Additionally, VPA rats displayed a greater bias toward exploring familiar objects, suggesting impaired novelty exploration ability, while the discrimination index significantly increased in the tDCS group (*p* < 0.05), and the time spent exploring new objects also increased in the tDCS group, indicating that tDCS improved object novelty exploration (Figures 2K, L).

### 3.5 Cognitive flexibility test

During CMRST, one rat in the CON group was excluded due to electrode detachment, while three rats each in the CON and VPA groups were eliminated because of electrode occlusion. So there were six rats in the CON group, six rats in the VPA group, and nine rats in the tDCS group. The results of CMRST indicated that during Stage 3, VPA rats required more trials to reach the learning criterion and made more errors compared to the CON group. In contrast, tDCS rats learned more quickly than VPA rats, suggesting that tDCS helped to alleviate learning deficits (Figures 2M–O). The results demonstrated that, in Stage 4, made more errors than CON rats, while tDCS rats made fewer errors compared to VPA rats, which suggests that tDCS helped to improve impaired cognitive flexibility (Figure 2P).

**FIGURE 3:**
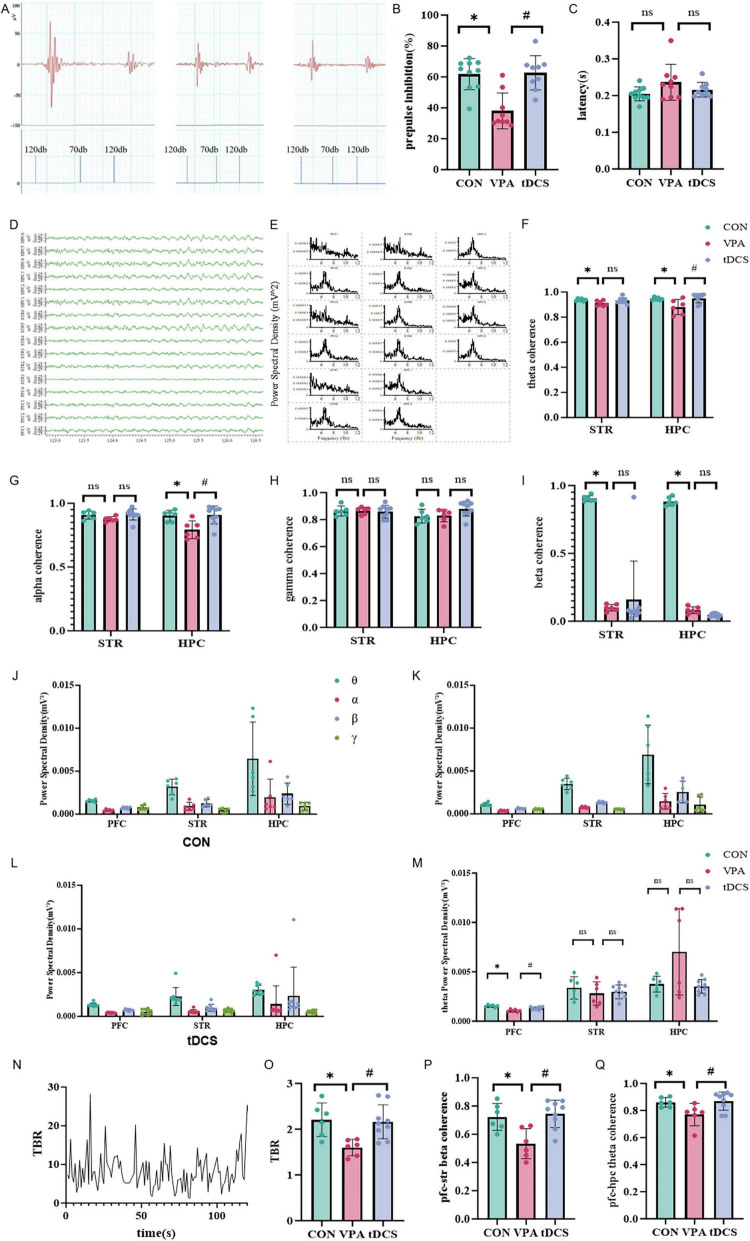
Startle reflex and electrophysiology recording results. **(A)** Startle reflex waveforms for each group of rats. **(B)** Results and analysis of the pre-pulse inhibition (PPI) index for each group (df = 2, H = 13.107, *p* = 0.001). **(C)** Results of the latency for each group (df = 2, H = 2.821, *p* = 0.244). **(D)** Local field potential (LFP) raw data. **(E)** LFP power spectrum distribution. PFC, prefrontal cortex; STR, striatum; HPC, hippocampus. **(F–I)** Coherence between frontal lobe and striatum and hippocampus in Cross-maze rule-shifting task (CMRST) waiting phase. **(F)** θ-band coherence, 4–8 Hz (STR: df = 2, H = 8.000, *p* = 0.018; HPC: df = 2, H = 8.035, *p* = 0.018). **(G)** α-band coherence, 8–12 Hz (STR: df = 2, H = 4.877, *p* = 0.087; HPC: df = 2, H = 7.614, *p* = 0.022). **(H)** γ-band coherence, 12–30 Hz (STR: df = 2, H = 0.433, *p* = 0.805; HPC: df _between_ _groups_ = 2, F = 2.445, *p* = 0.120). **(I)** β-band coherence, 30–80 Hz (STR: df = 2, H = 6.222, *p* = 0.045; HPC: df = 2, H = 7.380, *p* = 0.025). **(J–Q)** Whole LFP Analysis in CMRST, an example of theta/beta ratio (TBR) diagram and comparison of frontal TBR across groups and LFP coherence between prefrontal cortex (PFC) and STR, as well as between PFC and HPC, in each group of rats. **(J–L)** Power across frequency bands in frontal lobe, striatum and hippocampus of rats in each group. θ: 4–8 Hz, α: 8–12 Hz, β: 12–30 Hz, γ: 30–80 Hz. **(M)** Comparison of power in θ band among groups (PFC: df _between_ _groups_ = 2, F = 22.357, *p* < 0.001; STR: df _between_ _groups_ = 2, F = 0.551, *p* = 0.586; HPC: df = 2, H = 1.115, *p* = 0.573). **(N)** Transient TBR in the frontal lobe. TBR, theta beta ratio; θ: 4–8 Hz, β: 12–30 Hz. **(O)** TBR differences among the three groups, with the VPA group showing lower values (df _between_ _groups_ = 2, F = 6.725, *p* = 0.007). **(P)** Coherence in the β band between the frontal lobe and striatum for each group of rats (df _between_ _groups_ = 2, F = 8.994, *p* = 0.002). β: 12–30 Hz. **(Q)** Coherence in the θ band between the frontal lobe and hippocampus for each group of rats (df = 2, H = 6.372, *p* = 0.041). θ: 4–8 Hz. PFC, prefrontal cortex; STR, striatum; HPC, hippocampus. Data were analyzed by Kruskal-Wallis H test **(B,C,F,G,I,H_STR_,M_HPC_,Q)**, one-way analysis of variance (ANOVA) **(H_HPC_,M_PFC_,M_STR_,O,P)**. *p < 0.05, #*p* < 0.05. ns, no significant difference. In Startle reflex, *n* = 10 in the CON group, *n* = 9 in the VPA group, *n* = 9 in the tDCS group. In others tests, *n* = 6 in the CON group, *n* = 6 in the VPA group, *n* = 9 in the tDCS group.

Neither electrode detachment nor occlusion affected the Startle reflex measurements. Therefore, the final group sizes were maintained at 10 rats in the CON group, and nine rats each in the VPA and tDCS groups. The results of the Startle reflex (Figures 3A–C) indicated that there was no statistically significant difference in the latency of the startle reflex among the three groups of rats (*p* > 0.05), suggesting that there were no obvious abnormalities in the Startle reflex pathway of the VPA rats. Compared to the CON group, the PPI of VPA rats was significantly reduced (*p* < 0.05). However, the PPI of tDCS rats improved compared to VPA rats (*p* < 0.05), indicating that tDCS can ameliorate the PPI deficits in the VPA rats.

### 3.6 Electrophysiology recording results

**FIGURE 4:**
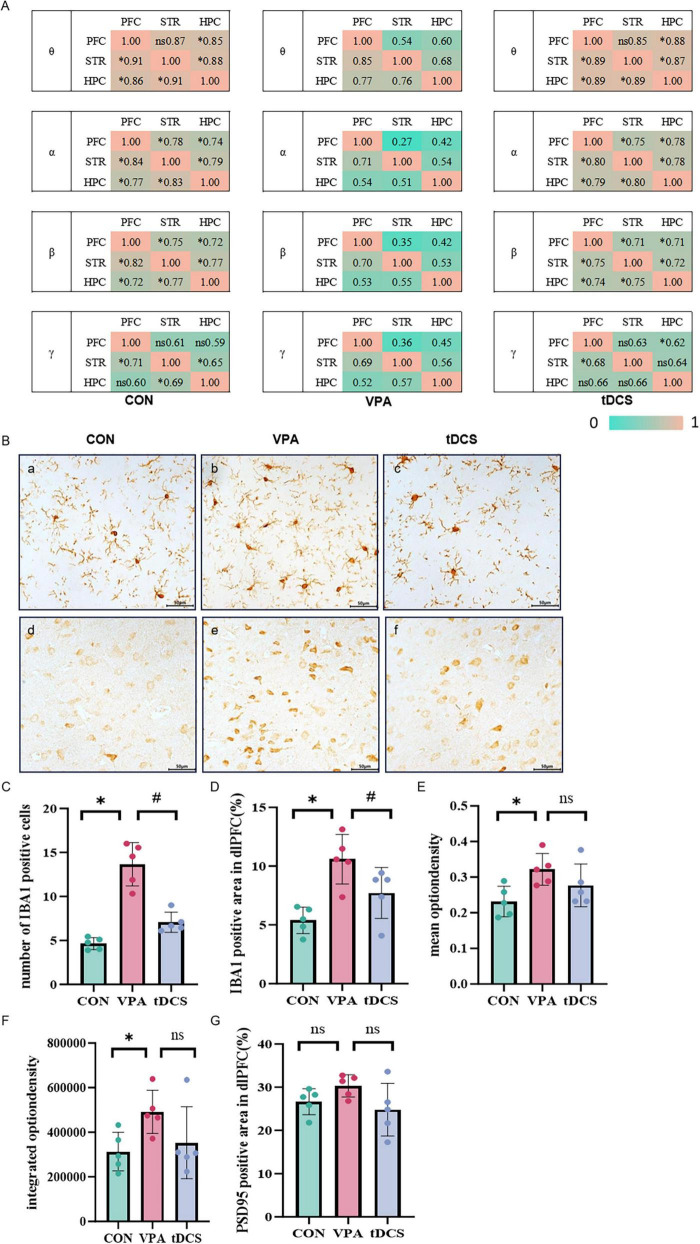
Immunohistochemical (IHC) results. **(A)** Coherence among prefrontal cortex (PFC), striatum (STR), and hippocampus (HPC) across various frequency bands in each group. Functional connectivity was significantly reduced in the valproic acid (VPA) group. Colder colors represent lower coherence values, while warmer colors indicate higher coherence values. θ: 4–8 Hz, α: 8–12 Hz, β: 12–30 Hz, γ: 30–80 Hz. Data were analyzed using one-way analysis of variance (ANOVA), comparing differences relative to the VPA group. *n* = 6 in the CON group, *n* = 6 in the VPA group, *n* = 9 in the tDCS group. **(B)** a–c, Immunohistochemical staining of microglia in three groups of mice. d–f, Immunohistochemical staining of PSD95 in three groups of rats. **(C)** Comparison of the number of microglia among the three groups of mice (df = 2, H = 12.500, *p* = 0.002). **(D)** Comparison of the results of the percentage of microglial cell area among the three groups of mice (df _between_ _groups_ = 2, F = 9.788, *p* = 0.003). **(E)** Comparison of mean optical density values of postsynaptic density protein 95 (PSD95) in three groups of rats (df _between_ _groups_ = 2, F = 4.127, *p* = 0.043). **(F)** Comparison of integrated optical density values of PSD95 in three groups of rats (df = 2, H = 6.180, *p* = 0.046). **(G)** Comparison of PSD95 positive area in three groups of rats (df _between_ _groups_ = 2.244, F = 2.244, *p* = 0.149). Data were analyzed by Kruskal-Wallis H test **(C,F)**, one-way analysis of variance (ANOVA) **(D,E,G)**. *p < 0.05, #*p* < 0.05. ns, no significant difference, *n* = 5 in the CON group, *n* = 5 in the VPA group, *n* = 5 in the transcranial direct current stimulation (tDCS) group. Scale bar = 50 μm.

As previously noted during the CMRST phase, successful LFP recordings were obtained from only six CON rats, six VPA rats, and nine tDCS rats after excluding subjects due to electrode detachment or occlusion (Figures 3D, E). LFP analysis during the waiting period in rats performing the CMRST (Figures 3F–I) indicated that functional connectivity between the frontal lobe, striatum, and hippocampus was generally lower in the VPA group than in the CON group. Additionally, more frequency bands of cerebral functional connectivity showed improvement in the tDCS group compared to the VPA group (*p* < 0.05). The comprehensive LFP analysis of each group revealed that the LFP θ-band power of frontal lobe was significantly lower in the VPA group than in the CON group (*p* < 0.05) (Figures 3J–M). Additionally, a decrease in both frontal-striatal β-band coherence (*p* < 0.05) and frontal-hippocampal θ-band coherence was observed (Figures 3P, Q). However, the θ-band power, β-band coherence between the frontal lobe and striatum, and θ-band coherence between the frontal lobe and hippocampus in tDCS rats increased compared to the VPA group (*p* < 0.05) (Figures 3J–Q). The coherence of the frontal lobe, striatum, and hippocampus across each frequency band revealed that functional connectivity within the brain network in the VPA group was significantly lower than in the CON group, particularly for connectivity from the frontal lobe to the striatum and hippocampus (Figure 4A). Following tDCS, the functional connections from the frontal lobe to the striatum and hippocampus in the tDCS group showed significant improvement compared to the VPA group (*p* < 0.05) (Figure 4A). The results indicated a decrease in frontal lobe function and its connectivity to the striatum and hippocampus in the VPA group, whereas tDCS improved both frontal lobe function and connectivity.

### 3.7 Immunohistochemistry

Following immunohistochemical staining (Figure 4B, a–c) (400 × ), the number of Iba1-positive cells was quantified for each group. Compared to the CON group, there was a significant increase in the number of Iba1-positive cells in the VPA group (*p* < 0.05). Following tDCS, the number of Iba1-positive cells in the tDCS group was significantly reduced compared to the VPA group (*p* < 0.05) (Figures 4C, D).

After immunohistochemical staining (400 ×, Figure 4B, d–f), the mean optical density value, integrated optical density value, and PSD95 positive area proportion were calculated for each group. Compared to the CON group, PSD95 positive expression was significantly increased in the VPA group (*p* < 0.05). However, following tDCS, there was no significant change in PSD95 positive expression compared to the VPA group (*p* > 0.05) (Figures 4E–G).

## 4 Discussion

The exact pathogenesis of ASD remains elusive, with accumulating evidence pointing to multifactorial etiology. Current research suggests that ASD may arise from intricate interactions among up to 1,000 susceptibility genes (De Rubeis et al., 2014), potentially modulated by environmental factors including toxins, infections and prenatal exposure to medications such as VPA (Berko et al., 2014). Epidemiological data consistently show that gestational VPA exposure substantially increases ASD risk in offspring (Prince et al., 2024), primarily through its ability to disrupt gene expression and chromatin remodeling during crucial neurodevelopmental periods. These molecular alterations can lead to abnormal embryonic gene and protein expression patterns, potentially resulting in neural tube closure defects and neurological dysfunction (Kuo and Liu, 2022). Mechanistically, as a histone deacetylase inhibitor, VPA exerts its effects by blocking lysine deacetylation in numerous protein targets, ultimately causing chromatin structural abnormalities and transcriptional dysregulation that may underlie both teratogenic effects and cellular toxicity (Larner et al., 2021). Given its ability to recapitulate core features of ASD, the VPA-induced rat model has emerged as an indispensable preclinical tool for studying disease mechanisms and testing novel therapeutic approaches (Chaliha et al., 2020).

We performed comprehensive behavioral phenotyping of the VPA-induced ASD rat model, assessing core behavioral domains including RRBs (wood chew and marble burying tests), social interaction (three-chamber social test), novelty exploration (novel object recognition test) and anxiety-like behaviors (open field test). In the wood chew test, VPA-exposed rats demonstrated significantly elevated chewing activity relative to controls (CON), as quantified by both greater mass loss of wooden sticks and more pronounced bite marks (*p* < 0.05). The marble burying test, a validated measure of rodent repetitive behavior (Partridge et al., 2023), revealed that VPA rats buried significantly more marbles than CON animals (*p* < 0.05), further validating the RRB phenotype. The three-chamber social test revealed pronounced sociability deficits and impaired social novelty preference in VPA rats. During Stage 2, VPA rats showed reduced sniffing time toward the social stimulus (S1) (*p* < 0.05) and lower social interaction indices compared to controls (*p* < 0.05). In Stage 3, VPA rats exhibited preferential interaction with the familiar mouse (S1) rather than the novel social stimulus (S2), with significantly reduced social novelty preference indices (*p* < 0.05), indicating substantial social communication impairments. Anxiety-related behaviors were quantified in the open field test (Sturman et al., 2018), where VPA rats spent significantly less time in the central zone than controls (*p* < 0.05). The novel object recognition test was used to assess recognition memory, with a discrimination index > 50% indicating novelty preference and < 50% reflecting familiarity preference (Naewla et al., 2019), which revealed significantly lower discrimination indices in VPA versus CON rats (*p* < 0.05), indicating recognition memory deficits. Extensive literature supports that intraperitoneal VPA administration at E12.5 effectively induces ASD-like phenotypes in offspring, demonstrating robust face and construct validity (Schneider and Przewłocki, 2005; Xiong et al., 2023; Zohny et al., 2023). Our VPA model rats showed tail malformations, increased RRBs, social deficits, heightened anxiety and recognition memory impairments, all showing statistical significance versus controls. These robust, quantifiable behavioral abnormalities confirm successful model establishment and provide a solid foundation for subsequent therapeutic interventions.

As one of the core symptoms of ASD, RRBs significantly impair functional behavior development and social skill acquisition in affected children. To quantitatively assess RRBs in our model, we employed two well-validated behavioral paradigms: the marble burying test and wood chewing. Following tDCS intervention, the tDCS group demonstrated marked reductions in both marble burying behavior (*p* < 0.05) and wood chewing activity (*p* < 0.05) compared to VPA rats, indicating significant amelioration of RRBs-like phenotypes. The three-chamber social test revealed substantial improvements in sociability following tDCS intervention. Compared to VPA rats, tDCS-treated rats showed significantly increased social interaction indices (*p* < 0.05) and enhanced social novelty preference indices (*p* < 0.05), collectively demonstrating improved social communication abilities. The novel object recognition test revealed that tDCS treatment significantly elevated the discrimination index (*p* < 0.05), with tDCS rats spending proportionally more time investigating novel objects, suggesting enhanced recognition memory function. Interestingly, while tDCS showed robust effects on RRBs and social-cognitive measures, we observed no statistically significant difference in center zone duration during open field testing between tDCS and VPA groups, suggesting that the current tDCS protocol may not significantly modulate anxiety-like behaviors in VPA rats. Whether tDCS can ameliorate anxiety-like behaviors warrants more in-depth investigation in future studies.

Cognitive flexibility is closely associated with the development and manifestation of core symptoms in ASD. Most experimental paradigms used to assess cognitive flexibility rely on behavioral outputs (Braff et al., 2001). We evaluated the cognitive flexibility of rats by CMRST and startle reflex. During the CMRST, VPA rats required significantly more trials than CON rats to reach the learning criterion during the response discrimination phase (initial rule acquisition) (*p* < 0.05), demonstrating impaired learning capacity. During the shift-to visual-cue phase, VPA rats persistently adhered to the previously learned strategy, repeatedly entering incorrect arms and committing more errors than CON rats, indicating marked deficits in set-shifting ability and reduced cognitive flexibility (*p* < 0.05). Notably, the performance gap between VPA and CON groups was more pronounced during the shift-to visual-cue phase compared to the response discrimination phase. Following tDCS intervention, treated rats showed fewer required trials to achieve the initial learning criterion compared to VPA rats, suggesting improved learning ability and significantly reduced errors during the shift-to visual-cue phase, demonstrating enhanced cognitive flexibility and set-shifting capacity (*p* < 0.05). These findings collectively indicate that tDCS effectively ameliorates cognitive flexibility impairments in ASD model rats.

The startle reflex represents a rapid defensive response to threatening stimuli, serving as a crucial neurophysiological mechanism for environmental adaptation. This protective response enables organisms to filter irrelevant sensory information and suppress competing behavioral reactions, thereby facilitating central nervous system processing of biologically significant stimuli (Braff et al., 2001). The startle reflex latency, operationally defined as the temporal interval between acoustic stimulus onset and reflex manifestation, provides a quantitative measure of conduction velocity within the neural pathways mediating this protective response. We found no statistically significant differences in the latency of the startle reflex among the three groups of rats, suggesting intact basic neural transmission pathways in ASD model rats (*p* > 0.05). However, our findings reveal significantly attenuated PPI in VPA rats compared to CON rats (*p* < 0.05), potentially attributable to impaired frontal inhibitory control. Studies confirm concurrent activation of both DLPFC and striatal regions during PPI tasks, with striatal dopamine dysregulation known to disrupt PPI (Swerdlow et al., 1992). Notably, studies have identified hyperactivation of dopamine D1 and D2 receptors in the dorsal striatum of VPA rats (Gandhi and Lee, 2020; Yoo et al., 2020), which may contribute to diminished top-down inhibition from frontal regions and consequent cognitive flexibility impairments. Meantime, attenuated PPI in VPA rats may also reflects impaired frontal-striatal circuit function, as PPI modulation involves coordinated activity across a distributed network including the PFC, thalamus, nucleus accumbens, and striatum (Li et al., 2009). Furthermore, pathological overactivation of glial cells in the PFC of individuals with ASD drives neuroinflammatory processes through excessive pro-inflammatory cytokine secretion, ultimately exacerbating neuronal toxicity and promoting cell death (Schalbetter et al., 2022). Concurrent reductions in prefrontal cerebral blood flow have been documented in ASD patients (Tang et al., 2022), potentially reflecting underlying metabolic deficits. These findings suggest that in ASD model rats, prefrontal cortical inhibitory function may be compromised by active glial-mediated neuroinflammation and cerebral hypoperfusion, impairing the ability to suppress irrelevant sensory input and consequently leading to cognitive flexibility deficits. Following tDCS intervention, the observed improvement in cognitive flexibility likely results from suppression of microglial overactivation and subsequent neuroinflammatory cascades, thereby restoring prefrontal inhibitory control. This neuro-modulatory effect may facilitate the recovery top-down regulation of sensory filtering processes, enabling more adaptive behavioral switching.

Functional connectivity represents the temporal coherence of neural activity patterns across anatomically distinct brain regions, serving as a key indicator of functional integration and network synchronization within the nervous system. Research has demonstrated that impaired cognitive flexibility is strongly linked to disrupted connectivity patterns within frontal-striatal circuits (Morris et al., 2016). Importantly, the integrity of these functional connections may correlate with the progression of cognitive flexibility impairments in ASD patients at different stages (Padron-Rivera et al., 2022). At the circuit level, the PFC plays a pivotal role in set-shifting tasks by continuously evaluating performance feedback, with its deep projection neurons maintaining continuous feedback transmission to the striatum, forming a critical neural substrate for flexible behavioral adaptation (Spellman et al., 2021).

The CMRST included both resting-state periods (absence of cognitive demands) and active task-engaged states. LFP recordings during the pre-task waiting period revealed significantly impaired functional connectivity between the PFC, striatum, and hippocampus in VPA rats across theta, alpha, and beta frequency bands compared to CON rats. These findings suggest that VPA rats exhibit fundamental deficits in prefrontal top-down regulation even before task initiation. LFP analysis throughout the entire task process demonstrated further electrophysiological divergence between groups, potentially attributable to the heightened salience of rewards-related feedback during the foraging phase. Notably, VPA rats showed markedly decreased prefrontal theta power versus controls and reduced theta/beta ratio (TBR), a validated index of cognitive processing (Picken et al., 2020). These electrophysiological alterations align with previous report demonstrating that prefrontal theta suppression is associated with impaired cognitive flexibility (Yeung et al., 2016). Our results strongly support the hypothesis that prefrontal hypofunction represents a core neural mechanism underlying cognitive flexibility impairments in this ASD model.

Cognitive function arises from dynamic network interactions across distributed brain regions rather than isolated activity in any single area (Just et al., 2004; Sporns et al., 2004). Notably, beta band functional connectivity between PFC and striatum critically regulates rewards prediction behaviors, whereas intact hippocampal-striatal connectivity is fundamental for cognitive flexibility (Su et al., 2024). Coherence analysis of LFPs revealed significantly widespread functional connectivity impairments in VPA rats compared to CON rats, with statistically significant decreases observed across theta (θ), alpha (α), and beta (β) bands (*p* < 0.05). Specifically, VPA rats exhibited markedly diminished prefrontal θ power (*p* < 0.05 vs. CON), attenuated beta band coherence between PFC and striatum (*p* < 0.05) and reduced θ-band coherence between PFC and hippocampus (*p* < 0.05). These results align with the observations of Courchesne and Pierce (2005), who reported under connectivity in long-range frontal interactions in individuals with ASD. These findings demonstrate impaired bidirectional connectivity in VPA rats, characterized by significantly attenuated top-down modulation from the PFC to both the striatum and hippocampus, compromised bottom-up connectivity from these subcortical structures back to the PFC. These findings collectively demonstrate impaired interregional information integration and deficient cooperative processing across neural networks, ultimately leading to cognitive flexibility deficits. Following tDCS intervention, compared to the VPA group, the tDCS group exhibited increased theta power (*p* < 0.05) in the PFC and significantly enhanced EEG coherence across brain regions, particularly between the PFC and the striatum as well as the hippocampus (*p* < 0.05). These findings suggest that tDCS may enhance PFC activation, improve frontal function, and primarily strengthen functional connectivity from the PFC to the striatum and hippocampus, thereby reinforcing top-down modulation by the PFC over these regions. Consistent with the results of the startle reflex, no differences were observed in startle latency among the groups. However, tDCS significantly ameliorated the reduced PPI in the VPA group, indicating that the impaired inhibitory capacity in VPA rats may not stem from abnormalities in the startle reflex pathway but rather from impaired modulation of downstream brain regions by the PFC.

The dorsolateral prefrontal cortex (DLPFC) serves as a critical neural substrate for cognitive control, integrating lower-level sensorimotor processes to enable goal-directed behavior (MacDonald et al., 2000). This higher-order association cortex plays multifaceted roles in working memory maintenance and manipulation (Barbey et al., 2013; Curtis and D’Esposito, 2003; Fuster, 2000), attentional set-shifting (Kondo et al., 2004), response inhibition and social cognition (Conson et al., 2015; McDonald et al., 2020), positioning it as a key interface between cognitive control and social information processing systems. These functional characteristics make the DLPFC represents a particularly promising therapeutic target for addressing core ASD symptomatology, where impairments across these domains are well-documented.

Microglia play crucial roles in both physiological and pathological neural processes, with M1 microglia exhibiting pro-inflammatory properties (increasing oxidative products and pro-inflammatory factors) while M2 microglia demonstrate anti-inflammatory and tissue-remodeling capacities (Liu et al., 2022). Under normal conditions, they support neuronal function through trophic supply, metabolic regulation and modulation of synaptic plasticity. However, in pathological states, activated microglia can release inflammatory cytokines and reactive oxygen species, contributing to neural dysfunction (Sierra et al., 2013). Emerging evidence indicates that tDCS can regulate the activity and function of microglia, with mechanism of action that may involve regulating the activation and secretion of microglia, thereby influencing neuroinflammation and oxidative stress responses (Saidi and Firoozabadi, 2021). In ASD specifically, aberrant activation and synaptic pruning of microglia are commonly observed, characterized by PFC microgliosis with M1 predominance over M2 phenotypes, alongside higher dendritic spine density (Luo et al., 2023; Schalbetter et al., 2022). Our experimental findings in VPA rats mirror these observations, showing significant increases in microglial number, activation state (evidenced by increased area fraction) and postsynaptic density protein 95 (PSD-95) expression in PFC compared to CON rats. PSD95, as the major scaffolding protein at glutamatergic synapses, critically regulates AMPA receptor anchoring and synaptic currents (Ziółkowska et al., 2023), with its aberrant expression potentially contributing to excitation-inhibition (E/I) imbalance in neurodevelopmental disorders like ASD (Keith and El-Husseini, 2008).

Notably, tDCS intervention produced sustained suppression of microglial activation and inflammatory markers in the PFC of VPA rats, despite showing minimal effects on PSD95 expression levels. Studies have demonstrated that direct current modulates microglial function by reducing migration capacity, suppressing the redistribution and organization of actin and β-tubulin (Cortese et al., 2014). Furthermore, it inhibits the expression of the Cacna1s subunit of type voltage-gated Ca^2+^ channels. Given that intracellular Ca^2+^ serves as a critical secondary messenger mediating multiple signaling pathways, blockade of Ca^2+^ channels can effectively suppress microglial activation (Lennikov et al., 2022). These findings suggest that impaired cognitive flexibility in VPA rats may stem from microglia overactivation, which disrupts normal synaptic pruning processes and leads to dysregulated PSD95 expression, ultimately causing excitation-inhibition imbalance. The therapeutic effects of tDCS appear to be mediated primarily through reducing microglia-derived inflammatory factors and neurotoxicity, although our current data did not demonstrate significant alterations in synaptic density. Further investigations are warranted to elucidate the precise mechanisms underlying these observations.

Accumulating evidence supports the therapeutic potential of DLPFC-targeted tDCS for improving cognitive flexibility, with studies demonstrating that cross-hemispheric tDCS over the DLPFC modulates task-switching ability (Leite et al., 2013; Tayeb and Lavidor, 2016). Our study establishes novel preclinical evidence for the efficacy of DLPFC-targeted tDCS in ASD model rats, with observed cognitive improvements significantly correlated with restored frontal-striatal/hippocampal functional connectivity and attenuated neuroinflammatory responses. This research paradigm bridges fundamental discoveries in cognitive neuroscience with translational therapeutic development, providing a targeted neuromodulation framework for ASD intervention. The unique position of DLPFC, as a brain region critically involved in cognitive control and social processing, makes it particularly promising for addressing ASD’s complex symptom. Future investigations should further delineate tDCS-induced neuroplasticity using multimodal imaging and identify predictive biomarkers of treatment response.

## 5 Conclusion

The proposed mechanism of tDCS-mediated cognitive flexibility improvement in ASD rats is as follows: In ASD rats, frontal lobe microglial overactivation (specifically M1 polarization) leads to neuroinflammation and oxidative stress. This disrupts the normal synaptic pruning function, resulting in PSD95 dysregulation and E/I imbalance. Consequently, frontal lobe function is impaired, with insufficient activation leading to reduced inhibitory capacity. When faced with multiple external information inputs, the rats are unable to suppress irrelevant information. Additionally, the functional connectivity between the frontal lobe and downstream striatum and hippocampus is weakened, preventing coordinated activity across brain regions and impairing the ability to flexibly switch behavioral patterns, ultimately leading to cognitive flexibility deficits. Following tDCS intervention, the activation level of microglia in the frontal lobe of VPA rats is reduced, improving the frontal lobe microenvironment. This reduction enhances inhibitory function of frontal lobe and strengthens top-down functional connectivity between the frontal-striatal and frontal-hippocampal pathways, promoting coordinated activity across brain regions and thereby ameliorating cognitive flexibility deficits.

## Data Availability

The raw data supporting the conclusions of this article will be made available by the authors, without undue reservation.
