# Supplementary material for: tDCS-induced enhancement of cognitive flexibility in autism: role of frontal lobe and associated neural circuits
Source: Front Behav Neurosci. 2025 Aug 12;19:1631236. doi: 10.3389/fnbeh.2025.1631236 (PMC12378128; doi:10.3389/fnbeh.2025.1631236)
Supplement: Supplementary file 1 [file Data_Sheet_1.docx]

Animals and methods

1.1 Electrode implantation

Six weeks after the birth of the offspring, the rats were anesthetized with isoflurane, and their heads were fixed using a brain stereotaxic apparatus, followed by exposure of the cranial surfaces of the rats. According to "The rat brain in stereotaxic coordinates"^[1]^，the screws positioned at coordinates (AP 4.5mm, ML 1mm, DV 0mm) and (AP -4mm, ML 3mm, DV 0mm) serve as grounding and reference points, respectively. For the stimulation electrode, the cathode and anode connection points are established using screws located at (AP 4.2mm, ML 2mm, DV 0mm) and (AP 1.6mm, ML 4.1mm, DV 0mm). Holes were made in the left dorsolateral prefrontal cortex (DLPFC; AP3mm, ML±3.2mm, DV-5.4mm), dorsal striatum caudate putamen (Cpu; AP-0.6mm, ML±2.6mm, DV4.2mm), hippocampus CA1 (AP-2.4mm, ML±2mm, DV3.2mm). LFP recording electrodes were attached to the openings, and the electrodes were fixed on the cranial surface using denture base tray resin.


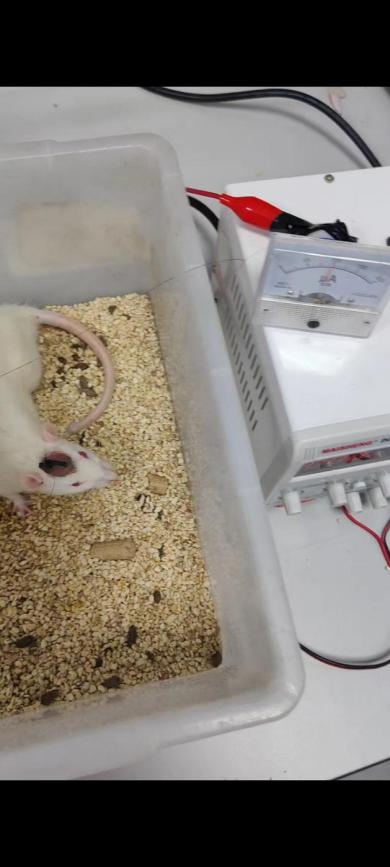


A.1 A.2


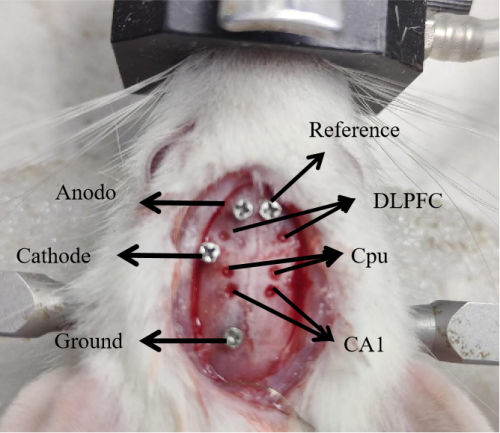


Fig.A Electrode implantation (A.1) Electrode implantation site. DLPFC: dorsolateral prefrontal cortex Cpu: caudate and putamen, CA1: hippocampal CA1 region (A.2) The process of administering tDCS.

1.2 Three-chamber social interaction test

The first phase of the Three-Chamber Social Interaction Test (TCT) is the habituation phase, which acclimatizes the tested rats to the experimental environment. This step is crucial to prevent alterations in the results of subsequent experiments. The second phase assesses whether the tested rats exhibit deficits in social interaction, since wild-type rats generally prefer to interact with other rats. The third phase evaluates whether the tested rats show deficits in social novelty, with wild-type rats preferring to interact with unfamiliar rats over ASD rats. Stranger rats consisted of healthy SD male rats that were similar in age to the tested rats and had never been housed with Stranger 1 (S1) or Stranger 2 (S2). These rats were randomly placed in metal cages situated in the two side chambers of a three-chamber box. The acclimatization period was set to 10 minutes for three consecutive sessions. After 30 minutes of acclimatization, if the rats no longer exhibited high levels of aggressive or potentially destructive behaviors (e.g., biting, incessantly climbing the wire cages, or excessive self-grooming), they were deemed suitable for use as social strangers; otherwise, the rats needed to be reselected for further training. Social preference index = (S1−E)/(S1+E)×100%. Social novelty preference index =(S2−S1)/(S2+S1) ×100%.

1.3 Marble burying test and Wood chew test

Marble burying test^[2]^ is mostly applied to assess repetitive stereotyped behaviors in autism. Briefly, a 5 cm layer of corn cob bedding was applied to the bottom of clean cages(50cm×35cm×20cm). The tested rats were put into the cage to adapt for 5min and then taken out. Twenty (5×4) black glass beads (16 mm in diameter) were evenly placed on the cage bedding, after which the rats were placed from the edge into the previously acclimatized cages for 5 min at liberty. Food and water were not provided during the test period. The number of beads whose buried volume exceeded 2/3 of the whole volume was counted.

Wood chew test^[3]^ was used to assess repetitive stereotyped behavior in rats. Each rat was housed in a separate cage equipped with fresh bedding material. with access to food and water ad libitum. Dried sticks of uniform shape and size were weighed and labeled. One stick was placed in each cage at 17:00 on the same day, and after overnight exposure, the sticks were taken out of the cages at 10:00 on the next day, and the feces were wiped off and put into an incubator at 37°C for drying overnight. After the drying process, the sticks were weighed once more. The mass difference between the initial and post-chewing measurements was calculated and recorded, representing the amount of stick material consumed by the rats.

1.4 Anxiety behavior test

Open field test^[4]^ was used to assess the level of anxiety in rats^[5]^. In Any-maze system, the open field box (80cm×80cm×50cm) was divided into 4×4 squares, the central 4 squares were defined as the center area, and the surrounding 12 squares were defined as the peripheral area, and the rats were gently placed in the center of the open field and allowed to move freely for 5 min, and the duration each rat spent in the central zone was measured and recorded.

1.5 Startle reflex

The startle reflex^[6]^ is a commonly used behavioral test that can be used to assess stress response, emotional state and cognitive ability in rats. The square Plexiglas box (13.5cm x 15.5cm x 27cm) with the speaker fixed on the top was fixed on the gravity sensor, the speaker was connected to the stimulator, and the gravity sensor signal was connected to the PowerLab 8. The speaker was tuned to 120db for 300ms during startle stimulation, and the speaker was set to 70db for 300ms during pre-stimulation in the double-pulse stimulation, which immediately followed the startle stimulation after an interval of 600ms. The double-pulse stimulation was turned on after the baseline was stable in an interval of 10-30s.

2. Result

2.1 Growth and development

Offspring injected with saline on day 12.5 of gestation served as the normal control group, and no tail deformities were observed in these rats. Offspring injected with VPA on day 12.5 of gestation constituted the autistic group and exhibited prevalent curved tail with varying degrees of deformity. The tail lengths were measured at weeks 2 and 3, revealing that rats in the VPA group had significantly shorter tails compared to those in the CON group. (P < 0.05) (Fig. B).

B


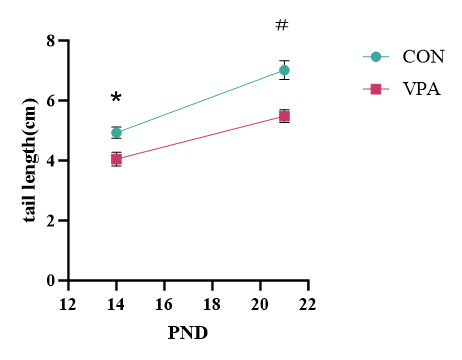


Fig.B Morphological changes in littermates. A comparison of changes in tail length was made between the normal control and autistic littermates (v=18, t=4.788, p<0.001). Statistical comparisons were performed using two-tailed independent samples t-tests, with significance set at *P<0.05, #*P*＜0.05. n=10 in the CON group and n=10 in the VPA group.

2.2 Repetitive stereotype behavior test


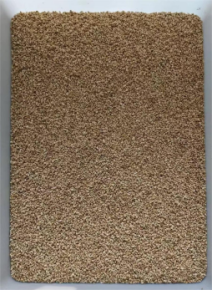

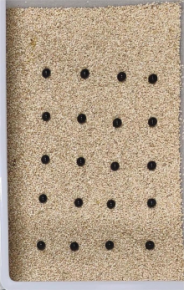

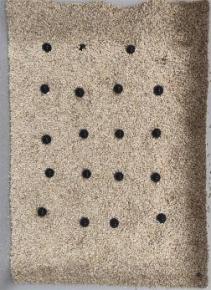

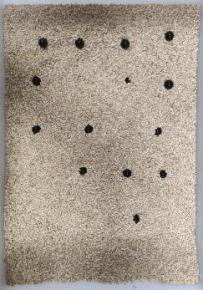

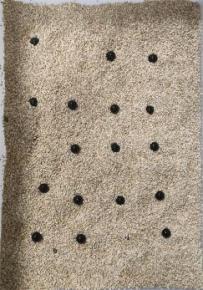


Adapt Start CON VPA tDCS

C.1


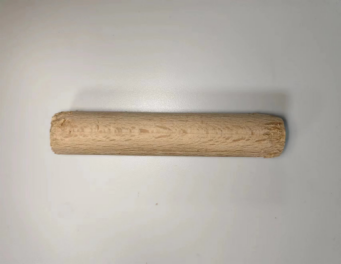

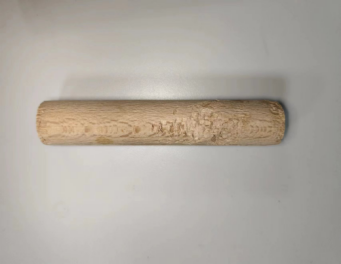

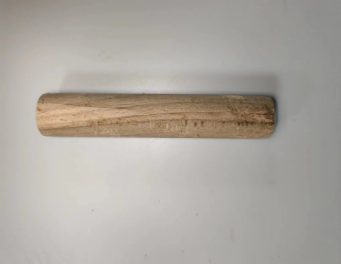


CON VPA tDCS

C.2

Fig. C Results of the marble burying test and wood chew test (C.1) Adaptation phase, start-up phase of the Marble burying test and the results of the three groups. (C.2) Chewed sticks.

2.3 Electrophysiology recording results

D


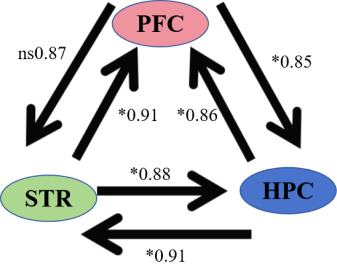

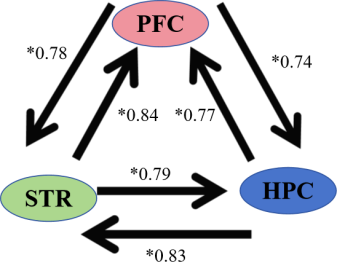

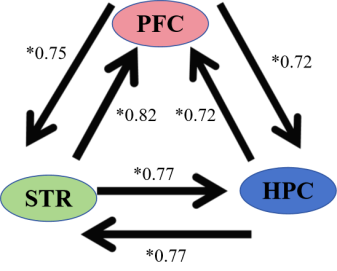

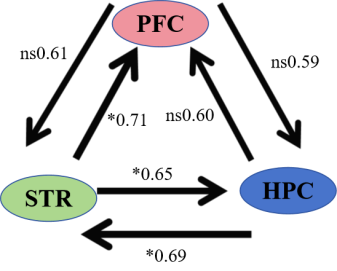

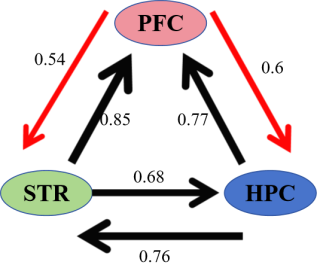

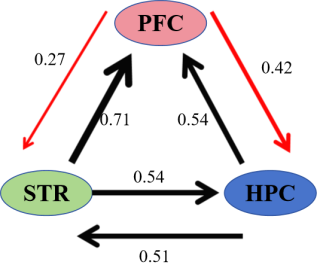

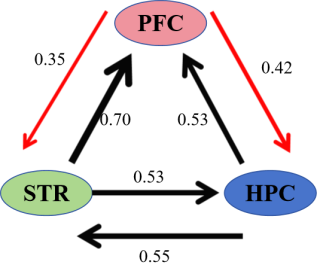

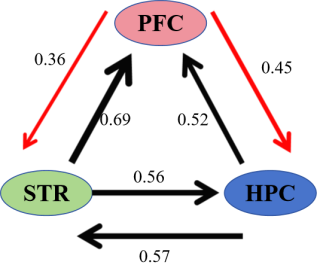

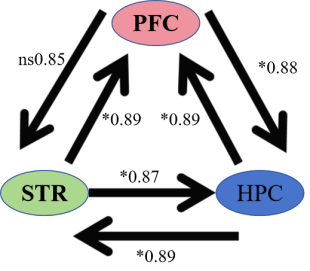

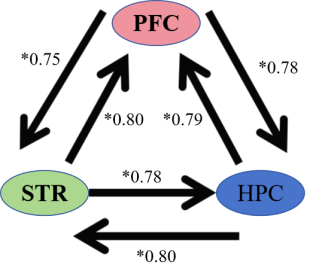

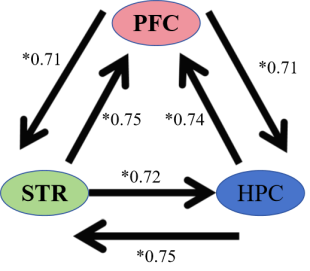

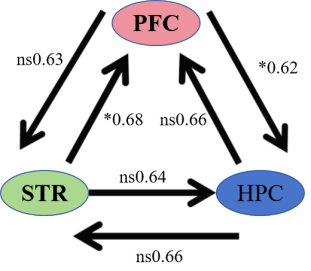


CON VPA tDCS

γ

θ

β

α

Fig. D Diagrams illustrating functional connections among PFC, STR and HPC in each group of rats. Functional connections from PFC to STR and HPC were significantly decreased in VPA group. θ：4-8Hz，α：8-12Hz，β：12-30Hz，γ：30-80Hz. Data were analyzed using one-way analysis of variance (ANOVA), comparing differences relative to the VPA group. *P < 0.05, ns: no significant difference. n=6 in the CON group, n=6 in the VPA group, n=9 in the tDCS group.

3. Discussion

E.1 E.2


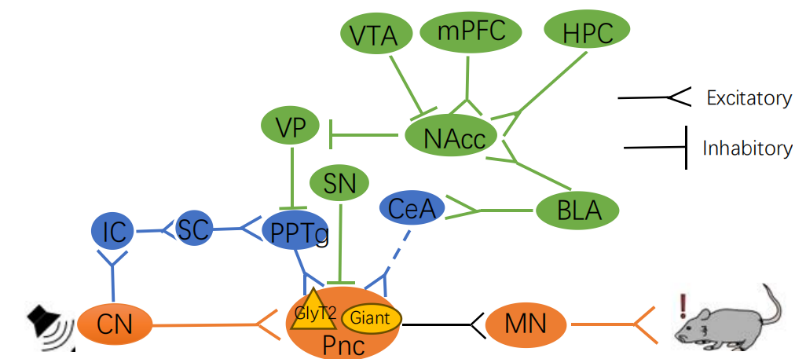

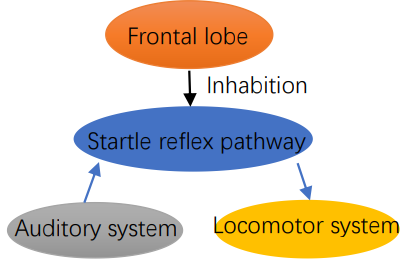


Fig. E Mechanism of PPI Regulation^[8]^. (E.1) The neural circuit involved in the acoustic startle response and prepulse inhibition (PPI). Primary auditory neurons activate the cochlear nucleus (CN), which relays auditory information to the caudal pontine reticular nucleus (PnC), ultimately activating motor neurons. During the PPI process (represented by the blue pathway), the prepulse inhibits the startle reflex by activating the inferior colliculus (IC), superior colliculus (SC), and the pedunculopontine tegmental nucleus (PPTg). The PPI pathway is also influenced by midbrain and limbic cortical structures (indicated by the light blue pathway). BLA: Basolateral amygdala; NAcc: Nucleus accumbens; VP: Ventral pallidum; HPC: Hippocampus; mPFC: Medial prefrontal cortex; SN: Substantia nigra; VTA: Ventral tegmental area; MN: Motor neurons; CeA: Central amygdala. (E.2) The mechanism of frontal lobe regulation of PPI, demonstrating how the frontal lobe enhances PPI by inhibiting the startle pathway.


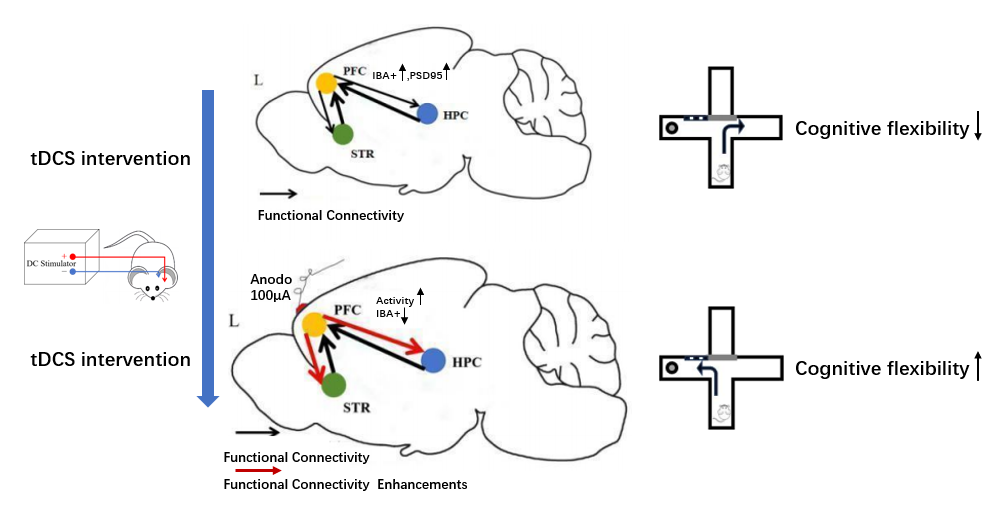


F

Fig.F Mechanism by which tDCS Improves Cognitive Flexibility in ASD Rats

**References**

[1] GEORGE PAXINOS C W. The rat brain in stereotaxic coordinates. 6th Edition [M]. New York: Academic Press, 2006

[2] SACAI H, SAKOORI K, KONNO K, et al. Autism spectrum disorder-like behavior caused by reduced excitatory synaptic transmission in pyramidal neurons of mouse prefrontal cortex [J]. Nat Commun, 2020, 11(1): 5140.

[3] WONG H, HOOPER A W M, NIIBORI Y, et al. Sexually dimorphic patterns in electroencephalography power spectrum and autism-related behaviors in a rat model of fragile X syndrome [J]. Neurobiol Dis, 2020, 146: 105118.

[4] STURMAN O, GERMAIN P L, BOHACEK J. Exploratory rearing: a context- and stress-sensitive behavior recorded in the open-field test [J]. Stress, 2018, 21(5): 443-52.

[5] ZHANG Y, GUO M, ZHANG H, et al. Lactiplantibacillus plantarum ST-III-fermented milk improves autistic-like behaviors in valproic acid-induced autism spectrum disorder mice by altering gut microbiota [J]. Front Nutr, 2022, 9: 1005308.

[6] CANO J C, HUANG W, FéNELON K. The amygdala modulates prepulse inhibition of the auditory startle reflex through excitatory inputs to the caudal pontine reticular nucleus [J]. BMC Biol, 2021, 19(1): 116.
